# Fellow-Workers and the Roll of Honour

**Published:** 1914-11-28

**Authors:** 


### ROUND THE WORLD.

[The Editor Invites Early Information and Contributions Marked "Fellow-Workers."]

### Fellow-Workers and the Roll of Honour.

The gallant Royal Army Medical Corps continues to suffer deplorable losses on the Continent, and it is very much to be regretted that in so-called civilised warfare the distinguishing marks of the military doctor have not served as a better protection. Indeed, terrible as may be the supposition, there appear to have been instances in which the sacred symbol of the Geneva Convention has particularly excited the ferocity of the enemy, and the Red Cross has attracted a torrent of lead instead of acting as a defence. At any rate, in the heat of action the Germans seem not infrequently to have been incapable of distinguishing combatants from non-combatants.

Captain Angus MacNab, who was attached as surgeon to the London Scottish Regiment, thus lost his life through the enemy being unable to respect his calling, for it has been reported by an onlooker that he was deliberately bayoneted in bright moonlight whilst stooping over a wounded comrade, in spite of the fact that his uniform was of a particularly distinguishing character. In private life Captain MacNab was well known in Harley Street, where he practised as an ophthalmic surgeon, having secured for himself by dint of sheer hard work a recognised position as an expert in diseases of the eye. A New Zealander by birth, he fulfilled an Arts course at Otago, and subsequently went to Edinburgh, where he graduated M.B., Ch.B. in 1901. Coming to London, he applied himself particularly to ophthalmology, becoming an F.R.C.S. Eng., and later supplemented his work by studies at Freiburg and Vienna. At the time of his death this keen and popular ophthalmologist held the appointments of ophthalmic surgeon to King Edward VII.'s Hospital at Windsor, chief clinical assistant to the Royal London Ophthalmic Hospital (Moorfields), and clinical assistant to the ophthalmic department at Charing Cross Hospital.

The younger generation of St. Mary's men are deeply lamenting the death of Lieutenant John Forbes O'Connell, who was recently killed in action on the Aisne. O'Connell, who qualified only two years ago by taking the M.B., B.S. Lond., was a first-class athlete and distinguished himself as captain of the hospital Rugby team. In January 1913 he was fifth in the R.A.M.C. entrance examination, and entered on his new work with great interest. However, his experiences of military surgery were not to be extensive, for, when the call came, he left for the Front with the Highland Light Infantry in the early days of the war, and within a few weeks fell at his post.

Lieutenant-Colonel Charles Dalton will be missed from the higher grades of the R.A.M.C., which he entered in 1901. An old Dublin student, he qualified by taking the conjoint Irish diplomas three years previously to his entering military service. It fell to Colonel Dalton to see a good deal of active service, for he had only been in the Army a short time before he took part in the Burma Expedition; whilst a few years later he served on the North-West Frontier with the Malakand Field Force (1897-8). The next two years again saw him campaigning, as he accompanied an expedition on the West Coast of Africa. His keen work on this occasion caused his name to appear in dispatches. So far he had come through scathless, but a year or two later he was badly wounded in South Africa whilst with the forces proceding to the relief of Ladysmith. For his services he was again mentioned in dispatches and awarded the Queen's medal with three clasps. Colonel Dalton's good fortune deserted him in the present war, and we have to deplore his death at the early age of forty-seven years.

A young "Guy's" man who was very popular in his year, Mr. Albert Evelyn Fairfax Kynaston, has succumbed to the rigours of active service, although not actually stricken down on the field of battle. Mr. Kynaston qualified M.R.C.S., L.R.C.P. ten years ago, and was known as an extremely good-natured colleague by all his fellow-workers at the hospital. A few years ago he joined the staff of the Poplar and Stepney Sick Asylum, where he studied until the outbreak of war, when he got a post in the Royal Naval Reserve. Being then appointed to H.M.S. *Devonshire*, within a few weeks he contracted enteric fever, which he successfully combated until pneumonia supervened and brought him down. He was buried at Dunstaith Naval Cemetery on Cromarty Firth.

Lieutenant Herbert Leslie Hopkins was, as a matter of fact, the first "Guy's" man to be killed on the Continent. Qualifying M.B., B.S. Lond. three years ago, he held the appointments of house physician to the Royal Infirmary at Derby and to the City of London Hospital for Diseases of the Chest. Subsequently he was for a time assistant county medical officer of health and tuberculosis officer at West Suffolk. Lieutenant Hopkins made practical use of his public health experience, which enabled him to take the M.D. Lond. in State Medicine last July. At the outbreak of war he obtained leave of absence to volunteer for service with the Forces, joining the R.A.M.C., and was made bacteriologist and pathologist to one of the military hospitals. However, he did not stay there long, as at the end of August he was sent abroad, and in a very short time had given up his life in the service of the wounded.

The sinking of H.M.S. *Hawke* deprived the Naval Medical Department of three capable officers, Staff-surgeon Ross, Surgeon Gustavus William Musgrave Custance, an old St. Thomas's man, and Mr. James Digby Watson, who had recently joined as temporary surgeon.

Mr. George Charles Cumberland Ross, senior medical officer on the *Hawke*, graduated in medicine as a student of Trinity College, Dublin, and joined the Navy two years after qualification in 1901. His promotion to the rank of Staff-surgeon followed in due course after eight years' service, and after various experiences he was appointed to the ill-fated cruiser at the beginning of August. His father is Colonel George Cumberland Ross, lately on the active list of the Indian Medical Service.

